# Dispersion as a waste-clearance mechanism in flow through penetrating perivascular spaces in the brain

**DOI:** 10.1038/s41598-021-83951-1

**Published:** 2021-02-25

**Authors:** Daniel E. Troyetsky, Jeffrey Tithof, John H. Thomas, Douglas H. Kelley

**Affiliations:** 1 Department of Mechanical Engineering, University of Rochester, Rochester, 14627 NY USA; 2 Department of Mechanical Engineering, University of Minnesota, Minneapolis, 55455 MN USA

**Keywords:** Biophysical models, Biological physics, Fluid dynamics, Computational biophysics

## Abstract

Accumulation of metabolic wastes in the brain is correlated with several neurodegenerative disorders, including Alzheimer’s disease. Waste transport and clearance occur via dispersion, the combined effect of diffusion and advection by flow of fluid. We examine the relative contributions of diffusion and advection in the perivascular spaces (PVSs) that surround penetrating cortical blood vessels and are filled with cerebrospinal fluid (CSF). To do so, we adapt prior analytic predictions of dispersion to the context of PVSs. We also perform advection-diffusion simulations in PVS-like geometries with parameters relevant to transport of amyloid-$\beta$ (associated with Alzheimer’s) in a variety of flows, motivated by in vivo measurements. Specifically, we examine solute transport in steady and unsteady Poiseuille flows in an open (not porous) concentric circular annulus. We find that a purely oscillatory flow enhances dispersion only weakly and does not produce significant transport, whereas a steady flow component, even if slow, clears waste more effectively.

## Introduction

**Figure 1:**
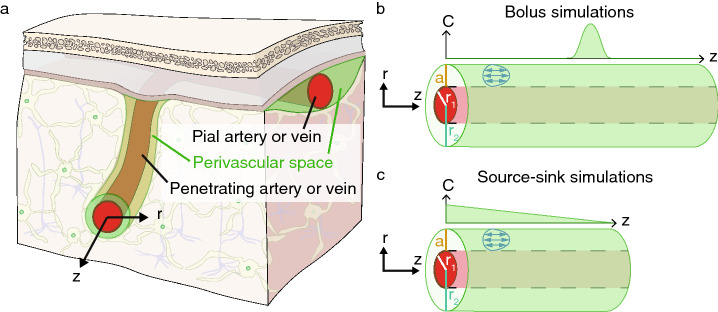
(**a**) A diagram depicting pial and penetrating arteries/veins in the brain and their surrounding perivascular spaces. (**b**, **c**) The idealized model (a concentric circular annular tube) of a penetrating perivascular space used in the simulations. Simulations are performed for a radial slice, on which the velocity field of a pulsatile flow with a net flow in the positive *z*-direction is depicted (blue arrows). The top plots in each panel are sketches of possible evolved solute concentration profiles corresponding to (**b**) bolus simulations and (**c**) source-sink simulations.

Cerebrospinal fluid (CSF) has long been recognized as a source of buoyancy and cushioning for nervous tissue, but some other physiological functions it may serve, such as removing metabolic waste, have been debated for decades^1^. One key question in this debate involves the nature of CSF transport in perivascular spaces (PVSs), which are annular channels surrounding blood vessels in the brain. As early as the 1980s, compelling evidence^2–4^ suggested that tracers injected into the CSF were transported into the brain along PVSs at rates that could not be explained by diffusion alone, implying advective transport by flowing CSF. More recently, interest in such questions has been renewed following in vivo observations of rapid transport of a tracer injected into the CSF by Iliff et al.^5^, who hypothesized that PVS flow constitutes a part of a pathway for clearance of metabolic waste from the brain, which they named the “glymphatic system.” While questions and controversies remain^6–8^, there is now ample evidence that CSF tracers are transported through PVSs at rates faster than possible with molecular diffusion alone^9–15^. In mice, an average CSF flow velocity of about 20 $\upmu$m/s has been measured in vivo in pial (surface) PVSs by injecting and tracking the motion of microspheres^9,10,15^. However, limitations in the resolution of magnetic resonance imaging and the penetration depth of two-photon microscopy have prevented direct measurements of flow speeds in PVSs surrounding penetrating arteries, which branch from pial arteries and dive deep into the brain (see Fig. 1a). Recent studies provide evidence of tracer motion consistent with advection in penetrating PVSs following stroke^14^, cardiac arrest^16^, and increased blood osmolality^17^. The nature of transport in penetrating PVSs under normal physiological conditions, however, is less certain. Furthermore, no study has yet presented CSF velocity measurements in penetrating PVSs.

In cases where experimental measurements are difficult or impossible, theory and simulations may offer novel insight and predictions. Fluid in penetrating PVSs that surround arteries, whose walls pulse with each heartbeat, almost certainly flows in a way that likewise oscillates at the heart rate. Some studies have argued that the traveling pulsations on artery walls also drive a mean flow, via a peristalsis-like mechanism known as “perivascular pumping”^18–21^. Experimental observations in surface PVSs have supported the idea^10^, but velocity measurements in penetrating PVSs remain unavailable. Other studies have argued that mean flow is unlikely or impossible^22–24^. It has also been pointed out that a purely oscillatory, zero-mean flow may nonetheless enhance transport and produce significant waste clearance in penetrating PVSs via Taylor dispersion^19,22,24–26^. That is, axial shear in the flow could cause strong radial concentration gradients and therefore rapid diffusion in the radial direction, so that the combined effects of advection and diffusion enhance the effective axial diffusivity along PVSs^27^. The term “Taylor dispersion” is usually applied to steady flows, but here we use it to indicate any enhancement of effective diffusivity caused by the combined action of shear flow and diffusion, for both steady and unsteady flows.

One of us has previously argued, on the basis of a simple qualitative model, that a purely oscillatory flow in PVSs cannot cause significant clearance, and that instead, some steady flow component is necessary^28^. This argument applies both to transport of tracers in the aforementioned studies and to transport of metabolic waste such as amyloid-$\beta$. Much prior work has focused on characterizing transport of amyloid-$\beta$ in the brain due to its hypothesized role in pathogenesis of Alzheimer’s disease^29^. It is generally accepted that CSF provides an important route for clearance of amyloid-$\beta$ from the brain^30^, and the rate of efflux via CSF has been estimated to be approximately the same as efflux across the blood-brain barrier^31^. In this paper we test the qualitative arguments presented earlier^28^ by carrying out numerical simulations of solute transport in a penetrating PVS (see Fig. 1a), idealized as a concentric circular annular tube (depicted in Fig. 1b,c). In the context of this study, we define solute clearance not as efflux from the entire perivascular network of the brain, but simply as clearance from the single PVS being simulated—a necessary first step toward brain-wide clearance. We use analytical expressions for velocity fields corresponding to steady, oscillatory, and pulsatile (steady plus oscillatory) laminar flows in the idealized PVS, driven by a prescribed pressure gradient. Quantifying the role of arterial pulsations in driving flow is outside the scope of this study, but we point out that our prescribed (steady and oscillatory) pressure gradients may be interpreted as arising from different driving mechanisms, including but not limited to arterial pulsations.

We solve the advection-diffusion equation numerically, considering two different configurations. In the first, we quantify the spreading of solute from a region of high concentration—a bolus—and its clearance from the PVS. This configuration models efflux of solute produced locally by a highly active brain region, allows the application of analytic dispersion predictions^27,32–34^, and is directly comparable to other recent studies modeling transport along PVSs^22,24,26,28^. In the second configuration, we quantify clearance along a PVS connecting a solute source to a solute sink. This configuration models clearance along PVSs between high-concentration regions and low-concentration regions, including PVSs around large veins connecting the deep brain to the subarachnoid space. In both configurations, our results strongly support the conclusion that a purely oscillatory flow produces significantly less clearance than even a slow mean flow.

## Methods

### Numerical simulations of advection and diffusion in a PVS

The transport of a passive solute in a flowing fluid is described by the advection-diffusion equation,

1 $$\begin{aligned} \frac{\partial C}{\partial t} + \mathbf{u}\cdot {{\varvec{\nabla }}}C= D\nabla ^2 C, \end{aligned}$$

where *C* is the solute concentration, *t* is time, $\mathbf{u}$ is the fluid velocity, and *D* is the diffusivity of the solute. Here we will consider only cases in which the velocity is known, so we need not be concerned with solving the Navier-Stokes equation that governs the flow. We shall consider purely axial laminar flows in a uniform PVS with cross-section in the form of a concentric circular annulus, with inner radius $r_1$ and outer radius $r_2$, as depicted in Fig. 1b,c. We use velocity fields that conserve fluid mass and satisfy the Navier–Stokes equation for a PVS of infinite length. These velocity fields are also valid for our finite tube because inertial effects are negligible and hence the entrance length for fully-developed flow is negligibly small (much less than the gap width *a*). The simulations are carried out for a finite length *L*. We consider flows driven by either a steady or oscillating axial pressure gradient *dp*/*dz* or a superposition of these flows (pulsatile flow); the flow velocity is then purely axial and axisymmetric, $\mathbf{u}= [0, 0, u(r,t)]$ in cylindrical coordinates $(r, \theta , z)$. Using the maximum concentration $C_0$ at time $t = 0$ as a concentration scale, the maximum flow velocity $u_{\text{max}}$ as a velocity scale, and the gap width $a \equiv r_2-r_1$ as a length scale, we define the following dimensionless quantities:

2 $$\begin{aligned} {\tilde{r}} = \frac{r}{a}, \quad {\tilde{z}} = \frac{z}{a}, \quad {\tilde{C}} = \frac{C}{C_0}, \quad {\tilde{t}} = t\frac{D}{a^2}, \quad {\tilde{u}}= \frac{u}{u_{\text{max}}}, \quad {\tilde{\nabla }} = a \nabla . \end{aligned}$$

The dimensionless form of Eq. (1) is then

3 $$\begin{aligned} \frac{\partial {\tilde{C}}}{\partial {\tilde{t}}} + Pe \, \tilde{{\mathbf{u}}} \cdot {\tilde{\nabla }} {\tilde{C}}= {\tilde{\nabla }}^2 {\tilde{C}}, \end{aligned}$$

where $Pe = u_{\text{max}} a/D$ is the Péclet number.

For pulsatile flow, the theory of Taylor dispersion states that an effective axial diffusion coefficient can be expressed as

4 $$\begin{aligned} D_{\text{eff}} = D(1 + R_{\text{s}} + R_{\text{o}}), \end{aligned}$$

where *D* is the molecular diffusivity of the solute and $R_{\text{s}}$ and $R_{\text{o}}$ are the enhancements to the diffusion coefficient from steady and oscillatory flow, respectively^35^. The dimensionless enhancement terms $R_{\text{s}}$ and $R_{\text{o}}$ are functions of both geometric and flow parameters, with results for steady Poiseuille flow in a circular pipe provided by Taylor^27^ and Aris^32^, and results for a concentric circular annular pipe in steady and oscillatory Poiseuille flows given by Aris^33^ and Tsangaris and Athanassiadis^34^, respectively.

To examine the transport of passive solutes within penetrating PVSs, we carried out numerical simulations of the advection-diffusion equation for axisymmetric, pressure-driven, laminar flows in the circular annular tube depicted in Fig. 1b,c. We treat the interior of the PVS as an open space rather than a porous medium. In vivo evidence shows that surface PVSs are open, not porous^10,36^. Less evidence is available for penetrating PVSs, but their observed eccentricity (misalignment of artery axis with PVS axis) would reduce hydraulic resistance and facilitate faster CSF flow only if they are essentially open spaces^36,37^. We would expect similar results in the case of Darcy–Brinkman flow with low to moderate Darcy number, that is, when the space is not entirely open, but porosity affects the flow only weakly. In the case of Darcy flow, where porosity dominates and viscosity plays no role, Taylor dispersion is eliminated altogether because the velocity does not vary radially.

We simulated dispersion due to steady flows, oscillatory (zero mean) flows, and pulsatile (steady plus oscillatory) flows, at Péclet numbers ranging from 0.1 to 10 to ensure that both diffusion-dominated and advection-dominated cases were considered. The diffusion coefficient of the solute and the geometric properties of the annulus remained fixed, and the peak fluid velocity $u_\text{max}$ was varied to change the Péclet number. All simulations were performed in Matlab, spatial gradients were calculated using a second-order central differencing scheme, and temporal derivatives were computed using a second-order Adams–Bashforth method. A Courant number of 0.2 was used for all simulations, and we ensured that our spatial and temporal resolution was adequate by verifying insensitivity to further refinement. Two different configurations were simulated, as described below.

### Analytical solutions

We use known analytical solutions for the velocity profiles as input to our simulations. Analytical solutions have been found for the velocity profiles and for the enhancements to the diffusion coefficients in both steady^33^ and unsteady^34^ laminar flows through a concentric circular annulus. Given the linear nature of the problem, these solutions can be summed to accurately approximate the diffusive enhancement of a pulsatile flow^35^. In each of the three cases, the velocity profiles are used as inputs to the simulations, and the outputs can be checked against analytic predictions of the enhanced diffusion coefficients. The analytical expressions for enhanced diffusion coefficients for steady flows were evaluated in Matlab; the more unwieldy expression for oscillatory flows was evaluated in Mathematica and is included as Supplementary File S1.

#### Steady flow

The velocity profile $u_{\text{s}} (r)$ for steady flow through a concentric circular annulus, driven by a constant pressure gradient ${dp_{\text{s}}}/{dz}$, is given by White^38^:

5 $$\begin{aligned} u_{\text{s}}(r) = - \frac{dp_{\text{s}}}{dz}\frac{1}{4\mu } \left[ (r_2^2 - r^2) - (r_2^2 - r_1^2)\frac{\ln {(r_2/r)}}{\ln {(r_2/r_1)}} \right] . \end{aligned}$$

The maximum velocity for this profile occurs at $r = r_{\text {max}} = \sqrt{(r_2^2-r_1^2)/ \left( 2 \ln {(r_2/r_1)} \right) }$ and is found to be

6 $$\begin{aligned} u_{\text{{s, max}}} = -\frac{dp_\text{s}}{dz}\frac{1}{4\mu } \left[ r_2^2 - \frac{r_2^2-r_1^2}{\ln {(r_2/r_1)}} \left( \frac{1}{2}+ \ln (r_2/r_{\text{max}}) \right) \right] . \end{aligned}$$

The corresponding dimensionless velocity is ${\tilde{u}}_{\text{s}} = u_{\text{s}}/u_{\text{{s, max}}}$ and the Péclet number is $Pe_{\text{s}} = u_{\text{{s, max}}} a / D$. An analytical solution for the enhancement term $R_{\text{s}}$ for lateral diffusion (see Eq. (4)) in such a flow is given by Aris^33^:

7 $$\begin{aligned} R_{\text{s}} = \kappa \frac{u_m^2(r_2^2-r_1^2)}{D^2} \end{aligned}$$

where $u_{\text{m}}$ is the mean flow speed and $\kappa$ is given by $\kappa = \kappa _{11} - 2\kappa _{12} + \kappa _{13}$ defined as

8 $$\begin{aligned} \kappa _{11}&= \frac{36(5 - 11\sigma ^2)\Sigma ^2 + 4(38 - 16 \sigma ^2 - 124\sigma ^4) \Sigma + 3(11 + 11 \sigma ^2 - 25 \sigma ^4 - 73 \sigma ^6) - 36 \sigma ^8\Sigma ^{-1}}{144(1 - \sigma ^2)S^2} , \end{aligned}$$

9 $$\begin{aligned} \kappa _{12}&= \frac{9(1 - 3\sigma ^2)\Sigma + (4 - 5\sigma ^2 -23 \sigma ^4) - 6\sigma ^6 \Sigma ^{-1}}{24(1-\sigma ^2)S} , \end{aligned}$$

10 $$\begin{aligned} \kappa _{13}&= \frac{1 - 3\sigma ^2 - 2\sigma ^4{\Sigma} ^{-1}}{8(1 - \sigma ^2)}, \end{aligned}$$

with $$\sigma = r_1/r_2$$, $$\Sigma = (1-\sigma ^2)/\ln {\sigma ^2}$$, and $$S = 1 + \sigma ^2 + 2\Sigma$$.

#### Oscillatory flow

For flow driven by an oscillatory pressure gradient $(dp_{\text{o}}/dz) \cos \left( {\omega t}\right)$, where $dp_{\text{o}}/dz$ is a constant amplitude and $\omega$ is the oscillation frequency, the oscillatory velocity profile has been determined analytically by Tsangaris and Athanassiadis^34^. Letting $\alpha = r_2 \sqrt{\omega /\nu }$ (where $\nu$ is the kinematic viscosity) and $\lambda = r_1/r_2$, the velocity is given by

11 $$\begin{aligned} u_{\text{o}}(r) = \frac{dp_{\text{o}}}{dz}\frac{i}{\omega \rho } \left[ F(r) -1\right] e^{i\omega t}, \end{aligned}$$

where $\rho$ is the density, $i=\sqrt{-1}$, and

12 $$\begin{aligned} F(r) = \frac{\left[ K_0(\lambda \alpha i^{1/2}) - K_0(\alpha i^{1/2})\right] I_0(r/r_2 \cdot \alpha i^{1/2}) + \left[ I_0(\alpha i^{1/2}) - I_0(\lambda \alpha i^{1/2})\right] K_0(r/r_2 \cdot \alpha i^{1/2})}{I_0(\alpha i^{1/2}) K_0(\lambda \alpha i^{1/2}) - I_0(\lambda \alpha i^{1/2}) K_0(\alpha i^{1/2})} \end{aligned}$$

here $I_0$ and $K_0$ are modified Bessel functions of the first and second kind, respectively. The maximum velocity is given by

13 $$\begin{aligned} u_{\text{{o, max}}} = \frac{dp_o}{dz}\frac{1}{\omega \rho }\left[ F(r_\text{max}) - 1 \right] . \end{aligned}$$

The corresponding dimensionless velocity is ${\tilde{u}}_{\text{o}} = u_{\text{o}}/u_{\text{{o, max}}}$ and the Péclet number is $Pe_{\text{o}} = u_{\text{{o, max}}} a / D$. The enhancement term $R_{\text{o}}$ for lateral diffusion (see Eq. (4)) for this velocity field is

14 $$\begin{aligned} R_{\text{o}}= \frac{\left( dp_o/dz\right) ^2}{{2(1+\beta )\omega ^4 \rho ^2 (r_2^2-r_1^2)}}\left[ r_2\left( G(r_2)\frac{\partial {\bar{F}}(r_2)}{\partial r} + {\bar{G}}(r_2)\frac{\partial F(r_2)}{\partial r} \right) - r_1\left( G(r_1)\frac{\partial {\bar{F}}(r_1)}{\partial r} +{\bar{G}}(r_1)\frac{\partial F(r_1)}{\partial r} \right) \right] , \end{aligned}$$

where $\beta = D/\nu$, the overbars denote complex conjugates, and *G*(*r*) is a function defined by Tsangaris and Athanassiadis^34^. (The expression for the function *G*(*r*) is quite lengthy and so will not be repeated here, but can be found in Supplementary File S1.)

#### Pulsatile flow

For pulsatile flow, the driving pressure gradient and velocity field can be taken to be superpositions of the steady and oscillatory cases: $dp_{\text{p}}/dz = - dp_{\text{s}}/dz - (dp_{\text{o}}/dz) \cos {(\omega t)}$ and $u_{\text{p}}= u_{\text{s}} + u_{\text{o}}$^35^. Accordingly, the enhancement to lateral diffusion is the sum of the steady (Eq. (7)) and oscillatory (Eq. (14)) enhancements. The velocity scale $u_{\text{{p, max}}}$ for such a flow is taken to be the sum of the amplitudes of the two components, so that $Pe_{\text{p}} = (u_\text{{s, max}} +u_{\text{{o, max}}}) a/D$. In our simulations for pulsatile flow, we take the amplitudes of the steady and oscillatory components to be the same, $u_{\text{{s, max}}} = u_{\text{{o, max}}} = \textstyle \frac{1}{2} \displaystyle u_\text{{p,max}}$.

### Parameter values

Parameter values for the geometry, fluid, flow, and solute used in the simulations were selected to be relevant for the transport of amyloid-$\beta$ in CSF through penetrating PVSs in the mouse brain. Geometric properties were adopted from the fit presented by Tithof et al.^37^ (Fig. 1f/Table 1f) to an image of an actual murine penetrating arterial PVS cross-section taken by Achariyar et al.^39^. The inner radius $r_1$ was taken to be the same as the measured radius of the actual arteriole, and the outer radius $r_2$ was calculated such that the cross-sectional area of the model PVS was the same as that of the actual PVS. The resulting values are $r_1$ = 7.21 $\upmu$m and $r_2$ = 20.2 $\upmu$m. Assumed values of the fluid density, $\rho = 993~{\text {kg/m}}^3$, and the kinematic viscosity, $\nu = 6.97\times 10^{-7}$ m$^2$/s, are those of water at 36.8 $^{\circ }$C^10^. The frequency of the oscillatory flows, $\omega = 10\pi$ rad/s, was selected to match the typical frequency of mouse arterial pulsations^10,20^, based on electrocardiography measurements demonstrating close correlation between velocity variations and the cardiac cycle^9,10^. The value of the diffusivity, $D=1.35\times 10^{-10}$ m$^2$/s, was chosen to be that of monomeric amyloid-$\beta$(1–42) in an aqueous solution^40^.

### Bolus simulations

In the first configuration simulated (Fig. 1b), we asked what would happen as a bolus evolved in each type of flow. That situation could provide a simple model of removal of waste produced deep in the brain. A Neumann boundary condition for zero radial flux, $\partial C/\partial r = 0$, was imposed at $r = r_1$ and $r = r_2$, so that no solute crosses the walls of the PVS. For computational convenience, a Dirichlet boundary condition for zero concentration, $C = 0$, was imposed at the inlet ($z = -L/2$) and outlet ($z = L/2$). The inlet and outlet conditions limit the applicable duration of our simulations, since we would expect nonzero concentration everywhere in the $t \rightarrow \infty$ limit. All results we present, however, are drawn from earlier times, when the concentration at the inlet and outlet is essentially zero, on the order of the numerical error. Thus our inlet and outlet conditions are practically equivalent to asserting $C = 0$ at $z = \pm \infty$. For simplicity, $C_0$ was set to 1 and hence *C* is a normalized concentration restricted to the range of values $0 \le C \le 1$. The initial concentration field of the bolus was prescribed as a Gaussian distribution

15 $$\begin{aligned} C(t=0) = \left\{ \begin{array}{ll} C_0\exp \left( {-\frac{1}{2} \left( \frac{z}{\gamma } \right) ^2} \right) , &{} \quad |z| \le w \\ 0, &{} \quad |z| > w \end{array} \right. \end{aligned}$$

with standard deviation $\gamma$. The parameter *w* was used as a cutoff distance from the center of the Gaussian, past which the initial concentration was fixed at zero. In each simulation, $\gamma = 2$ $\upmu$m and $w = 6$ $\upmu$m. Table 1 lists the values of the parameters used in each simulation. The case of pure diffusion was simulated using the same grid parameters as for oscillatory flow. Data was stored every 0.2 s of the simulation.

Net solute transport can be quantified by tracking the motion of either the center of solute mass or the axial location where radially-averaged concentration is highest. For Gaussian solute distributions, the center of mass coincides with the location of peak concentration. In this study, we tracked concentration peaks to evaluate net transport. Additionally, the percentage of solute to travel more than 250 $\upmu {\text {m}}$ from the center of the original concentration profile was quantified as a function of time. This distance was selected as a reasonable bifurcation-free length of a penetrating PVS^22,41^. The diffusive contribution to this transport is quantified using the full width of the mean concentration profile at half its maximum concentration (FWHM), which specifies the region containing $76\%$ of the solute (for a Gaussian distribution). FWHM grows more rapidly when dispersion is stronger, or equivalently, when the effective diffusion coefficient is larger.

**Table 1:** Parameter values for the bolus simulations.

Steady					
*Pe*	$u_{\text{{s, max}}}$ ($\upmu$m/s)	$u_{\text {m}}$ ($\upmu$m/s)	L ($\upmu$m)	$\delta z$ ($\upmu$m)	$\delta r$ ($\upmu$m)
0.1	1.039	0.685	5600	0.50	0.2598
1	10.39	6.85	4400	0.50	0.1299
5	51.96	34.25	3200	0.50	0.1299
10	103.9	68.5	3200	0.50	0.1299

Oscillatory					
*Pe*	$u_{\text{{o,max}}}$ ($\upmu$m/s)	$u_{\text {m}}$ ($\upmu$m/s)	L ($\upmu$m)	$\delta z$ ($\upmu$m)	$\delta r$ ($\upmu$m)
0.1	1.039	0	3200	0.50	0.1299
1	10.39	0	3200	0.50	0.1299
5	51.96	0	3200	0.50	0.1299
10	103.9	0	3200	0.50	0.1299

Pulsatile					
*Pe*	$u_{\text{{p, max}}}$ ($\upmu$m/s)	$u_{\text {m}}$ ($\upmu$m/s)	L ($\upmu$m)	$\delta z$ ($\upmu$m)	$\delta r$ ($\upmu$m)
0.1	1.039	0.3425	5600	0.50	0.2598
1	10.39	3.425	3200	0.50	0.1299
5	51.96	17.125	2800	0.50	0.1299
10	103.9	34.25	2800	0.50	0.1299

The domain length *L* was adjusted to ensure near-zero concentration at the inlet and outlet. In the cases of steady and pulsatile flows, this was done such that concentration near the boundaries remained near zero at least until the peak concentration was translated a distance of 300 $\upmu$m. As such, the domains of lower Pe flows were longer to minimize diffusive transport to the boundaries, which could lead to misleading results. Grid sizes $\delta z$ and $\delta r$ (in the axial and radial directions, respectively) were adjusted to ensure stability and convergence. $u_{\text {m}}$ is the mean velocity, averaged over both space and time.

The radially-averaged concentration, which varies axially and changes over time, is given by

16 $$\begin{aligned} C_m(z,t) = \frac{\int _{r_1}^{r_2} 2 \pi r C(z,r,t) \,dr}{\pi \left( r_2^2-r_1^2\right) }. \end{aligned}$$

We calculated $C_m(z,t)$ from simulation results. Then, for each time *t* in the simulation, we fit the radially-averaged concentration to an expression of the form

17 $$\begin{aligned} {\mathcal {C}}_{\text{m}}(z_1,t) = \frac{m_0}{\left( r_2^2-r_1^2\right) \sqrt{4 \pi ^3 k t}}e^{-z_1^2/4 k t} , \end{aligned}$$

where *k* is the sole fit parameter, $z_1 = z-z_{\text {peak}}$, $z_{\text {peak}}$ is the axial location of the concentration peak, and $m_0$ is the total mass of solute (unchanged from time $t=0$):

18 $$\begin{aligned} m_0 = \int _0^L \int _{r_1}^{r_2} 2 \pi r C(z,r,0) \,dr \,dz. \end{aligned}$$

This functional form was shown by Taylor^27^ to describe the dispersion of a Gaussian solute distribution by a steady flow, with *k* being the effective diffusivity. By fitting, we obtained a value for *k* for each time step in each bolus simulation, then took the effective diffusivity $D_{\text {eff}}$ in each simulation to be the average of *k* at all times after a convergence to $\pm 1\%$.

### Source-sink simulations

In the second configuration simulated (Fig. 1c), we set out to quantify clearance rates when a PVS connects a region of high solute concentration to a region of low solute concentration. This configuration could model multiple scenarios: transport of dextran along a penetrating arterial PVS after an intracisternal dextran injection or amyloid-$\beta$ transport along either the large veins that connect the deep brain to the subarachnoid space^42^ or perhaps penetrating venule PVSs that connect to pial vein PVSs. We again imposed the no-flux boundary condition $\partial C / \partial r = 0$ at $r=r_1$ and $r=r_2$. We made the inlet a source by imposing a $C=1$ boundary condition, and we made the outlet a sink by imposing $C=0$, again considering *C* to be a normalized concentration, $0 \le C \le 1$. The initial concentration field was $C_0 = 0$ throughout the domain. The domain length was fixed at $L=250 \, \upmu {\text {m}}$ with axial and radial grid sizes $\delta z=0.5 \, \upmu$m and $\delta r=0.1299 \, \upmu$m, respectively, for all simulations. Steady, oscillatory, and pulsatile simulations were performed with Péclet numbers 0.1, 1, and 10, with the same maximum and mean velocities as in the cases listed in Table 1. Data was stored every 0.05 s of the simulation.

From the maximum velocities reported in Table 1, the provided values for $r_1$ and $r_2$, and Eq. (6), the amplitudes of the pressure gradients required to drive each steady flow can be determined to be 33.16 Pa/m, 331.6 Pa/m, 1658 Pa/m, and 3316 Pa/m for Pe = 0.1, 1, 5, and 10, respectively. Because the Womersley number is small, at any given time the velocity profiles for the oscillatory and pulsatile flows are nearly identical to that of a steady Poiseuille flow. As such, these pressure gradients also correspond to the maximum pressure gradients in the oscillatory and pulsatile flows. The source of these pressure gradients remains a subject of debate and will not be discussed here.

We quantified efflux by measuring $d\phi (t)/dt$, where $\phi (t)$ is the normalized mass of solute transported through the domain. Normalized mass was used for consistency with the normalized concentration used in simulations. This mass flux was calculated as

19 $$\begin{aligned} \frac{d\phi (t)}{dt} = \int _{r_1}^{r_2} 2 \pi r \left( C(z,r,t)u(r) - D \frac{dC(z,r,t)}{dz}\right) dr, \end{aligned}$$

where $z = 125\,\upmu$m is the cross-section at which this calculation is performed and *u*(*r*) is the velocity profile of the flow which was either steady, oscillatory, or pulsatile. For oscillatory and pulsatile flows a mean transport rate was calculated by averaging the flux over an oscillation period.

## Results

### Bolus simulations

**Figure 2:**
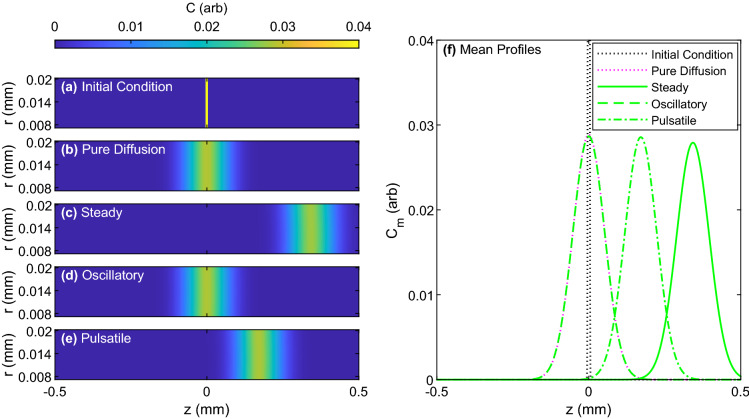
Snapshots of solute concentration fields for three different flow types with $Pe= 5$ at $t = 10$ s. (**a**–**e**) Concentration fields for (**a**) the initial condition, (**b**) pure diffusion, and (**c**) steady, (**d**) oscillatory, and (**e**) pulsatile Poiseuille flow. (**f**) Radially-averaged concentration profiles for the initial condition and steady, oscillatory, and pulsatile Poiseuille flow. Flows with faster steady components exhibited greater displacement of the peak concentration, as well as slightly lower peaks and wider solute distributions. Note that the initial condition shown in panels (**a**, **f**) has a peak concentration of 1 which extends beyond the colorbar/y-axis limit of 0.04. An animation of this figure, without the pure diffusion case, is provided in Supplementary Video 1.

Example concentration fields and axial mean concentration profiles for pure diffusion and steady, oscillatory, and pulsatile Poiseuille flows with $Pe = 5$ are depicted in Fig. 2. An animation of the transport for the three flow types is provided in Supplementary Video 1; the cases of $Pe=1$ and $Pe=10$ are also included as Supplementary Videos 2 and 3, respectively. Figure 2, as well as the Supplementary Videos 1, 2 and 3, make clear some important points regarding the solute transport: (1) over a given time interval, the concentration peak is displaced farthest by steady flow (Fig. 2c,f). (2) The concentration peak is not displaced at all by oscillatory flow (Fig. 2d,f). (3) The pulsatile flow (Fig. 2e,f) displaces the peak half as far as the steady flow (Fig. 2c,f), because the mean flow is half as fast at the same Péclet number and the oscillatory component causes no displacement. (4) The concentration peak is highest in the case of oscillatory flow (Fig. 2f). (5) The concentration peak for steady flow is slightly lower than that of pulsatile flow, which is slightly lower than that of oscillatory flow (Fig. 2f).

**Figure 3:**
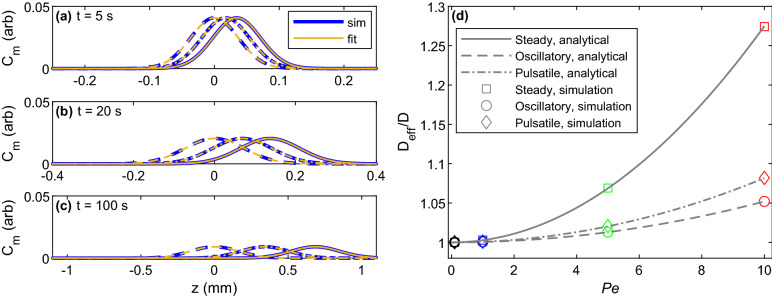
Snapshots of fitted concentration curves for steady, oscillatory, and pulsatile flows (continuous, dashed, and dot-dash curves, respectively) with $Pe = 1$ at (**a**) $t = 5$, (**b**) $t = 20$, and (**c**) $t = 100$. Note that (**a**–**c**) have different limits in *z*. (**d**) Effective diffusion coefficients from analytical expressions and numerical simulations. Points are colored by Péclet number: black for $Pe=0.1$, blue for $Pe=1$, green for $Pe=5$, and red for $Pe=10$. The discrepancy between simulations and analytical solutions does not exceed 0.05%.

We computed the effective lateral diffusion coefficient for each simulation using Eq. (17) and compared it to the analytical prediction given by Eqs. (4), (7), and (14). Examples of fitted curves are shown alongside mean concentration profiles for each flow type at $Pe=1$ in Fig. 3a–c. In each case shown in the figure, the coefficient of determination ($R^2$) for the fit was at minimum 0.9999. Although Fig. 3a–c shows only the $Pe=1$ case, the accuracy of the fit is comparable for other flows. The resulting effective lateral diffusion coefficients are plotted in Fig. 3d. For all cases studied, the effective diffusion coefficients from the simulations matched the analytical predictions to within 0.05%, validating the simulations. Steady Poiseuille flow gives rise to a larger effective diffusivity than oscillatory or pulsatile flow for all Péclet numbers evaluated. As *Pe* approaches 0, the enhancement ratio $D_{\text{eff}}/D$ approaches 1. Significantly, the largest effective diffusivity obtained (at $Pe = 10$) was only $\sim 27$% greater than pure diffusion, in contrast to the near doubling reported by Asgari et al.^22^ and attributed to the oscillatory component of the flow. The largest enhancement in effective diffusivity we found for oscillatory flow was 5.2%, at $Pe = 10$.

**Figure 4:**
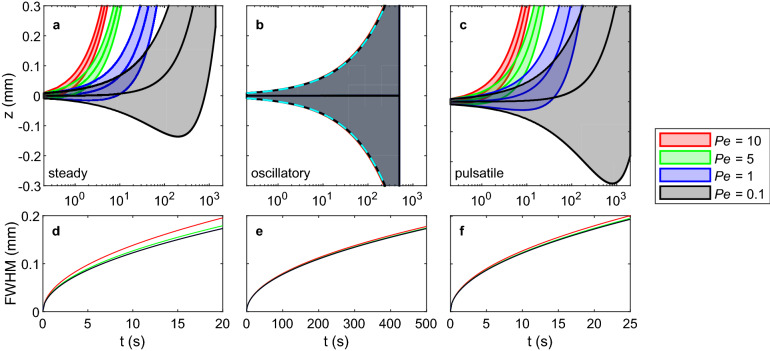
Transport of the solute in the bolus simulations for (**a**, **d**) steady flow, (**b**, **e**) oscillatory flow, and (**c**, **f**) pulsatile flow, at different Péclet numbers. In (**a**–**c**), the center curves indicate the axial position of the peak axial mean concentration, and the upper and lower curves indicate axial positions where the local concentration is half of the maximum concentration. Peaks move at constant speed, although their trajectories appear as curves on the semi-logarithmic axes used to accommodate the very different timescales for flows of different Péclet number. The full width at half-maximum (FWHM), defined as the distance between the upper and lower curves, and is plotted in (**d**–**f**). The rate of increase of the FWHM slows continuously in all cases. In (**b**), all cases have nearly identical FWHM and are shown in the same manner as (**a**, **c**). The dashed light blue curves depict the analytical FWHM for pure diffusion. For all three flow types, the FWHM is greatest for the case with highest Péclet number. Curves for $Pe = 0.1$ and $Pe = 1$ nearly overlap. Note that the horizontal axis in each plot depicts time, but the limits for the FWHM plots are truncated to the duration of the shortest simulation for each flow type.

The location and width of high-concentration regions varied over time, as shown in Fig. 4. In each case, the concentration peak traveled at a rate corresponding to the mean flow velocity (which is zero in the case of oscillatory flow). In flows with an oscillatory component, there was slight oscillatory movement of the concentration peak at a frequency coinciding with the frequency of flow oscillation. Solute spreading, as quantified by FWHM, increased noticeably with Péclet number in steady and pulsatile flows. In purely oscillatory flows, increasing *Pe* caused only slightly faster spreading. It is particularly useful to compare the case of steady flow with $Pe = 5$ in Fig. 4a with the case of pulsatile flow with $Pe = 10$ in Fig. 4c, as these two flows have the same $u_{\text{{s, max}}}$. The locations of their concentration peaks never differ by more than 0.001%. Their FWHM values are similar and converge over time, differing by just 0.6% at $t = 25$ s.

For cases with a nonzero steady component of flow (i.e., steady and pulsatile flows), we would expect that sharp gradients in the initial concentration would cause solute transport in the direction opposite the flow at early times, when $|{\tilde{\nabla }}^2 {\tilde{C}}| > |Pe\, {\tilde{u}} \cdot {\tilde{\nabla }} {\tilde{C}}|$. As shown in Fig. 4a, upstream transport is clearly evident in the $Pe = 0.1$ and subtly noticeable in the $Pe = 1$ case. We expect it would also be observable in the $Pe = 5$ and $Pe = 10$ cases if we increased the temporal resolution at early times. Upstream transport is a transient, however, inevitably overwhelmed at later times.

**Figure 5:**
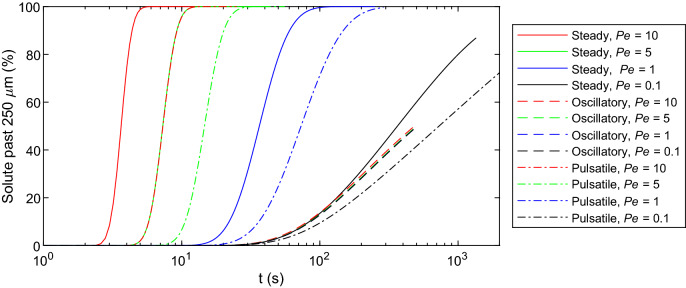
Clearance times for a given percentage of total solute to travel axially more than 250 $\upmu {\text {m}}$ to the right, from the center of the initial bolus of solute. Steeper slopes indicate more effective solute clearance. For oscillatory flows, transport in both directions ($\pm z$) was included, meaning effective directional transport is half the value plotted here.

Clearance times are depicted in Fig. 5. In steady and pulsatile flows, the time necessary to clear a given percentage of solute varies nonlinearly with both *Pe* and time. The curves are consistent with transport across the boundary being generally advection dominated, with the rate of transport increasing until the peak concentration moves across the measurement boundary $z=250\,\upmu$m, after which the transport rate decreases. Because the instant at which 50% of the solute is cleared also coincides with the peak concentration passing $z=250\,\upmu$m, transport during earlier and later times is respectively enhanced and hindered by diffusion. Hence, the approach to 100% is slower than the departure from 0%, but the curves appear approximately symmetric only because the axes are semi-logarithmic. For all three types of flows, lower *Pe* values lead to longer times for a given percentage of solute to be cleared. However, for all cases of oscillatory flow, clearance times were comparable to each other and longer than that of the $Pe = 0.1$ steady flow case. Importantly, we computed the clearance times for oscillatory flows using transport in both directions ($\pm z$), meaning the effective directional transport is half the plotted value (and hence slower than the pulsatile flow). Together, these findings suggest that a small steady flow is capable of enhancing transport substantially more than an oscillatory flow of 100 times greater amplitude.

### Source-sink simulations

**Figure 6:**
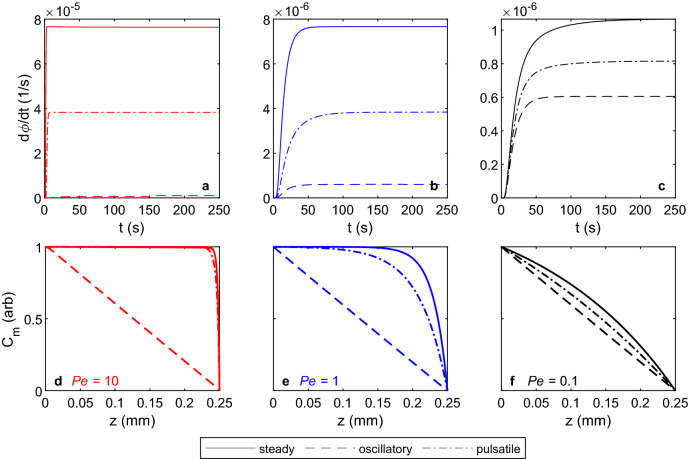
(**a**–**c**) Rates of normalized solute mass efflux from the domain in the source-sink simulations and (**d**–**f**) radially-averaged asymptotic solute concentration profiles (plotted at $t=250$ s) for flows with (**a**, **d**) *Pe* = 10, (**b**, **e**) *Pe* = 1, and (**c**, **f**) *Pe* = 0.1. Flux was calculated across the mid-plane of the domain and, by conservation of mass, must be equal to the efflux from the domain in the asymptotic state. Increasing *Pe* leads to greater rates of solute efflux for steady, pulsatile, and oscillatory flows, as well as a greater difference between concentration profiles for oscillatory and steady/pulsatile flows. The profiles shown in (**d**–**f**) can be compared to the sketch in Fig. 1c, which illustrates diffusion-dominated transport. Animations that include panels (**d**–**f**) are provided in Supplementary Videos 4, 5 and 6, respectively.

Rates of solute clearance in source-sink simulations for $Pe=\{10, 1, 0.1\}$ are illustrated in Fig. 6a–c, which show the rate of normalized mass $\phi (t)$ of solute cleared in each simulation, varying over time. After an initial transient, all three flows at all three Péclet numbers exhibit a constant rate of solute clearance. As in the bolus simulations, clearance is dominated by the steady component of the flow, which is strongest for steady flow, weaker for pulsatile flow, and zero for oscillatory flow. (Recall that we define *Pe* for pulsatile flow in terms of the maximum velocity, resulting from steady and oscillatory components of equal magnitude.) It is evident that the asymptotic clearance rates for steady and pulsatile flows decrease by a factor of 10 when *Pe* is changed from 10 to 1 (Fig. 6a,b; note the difference in the *y*-axis limits). Furthermore, for fixed $Pe=10$ or $Pe=1$, the asymptotic solute clearance rate for the pulsatile flow is half of that of the steady flow. However, for $Pe=0.1$ in which diffusion dominates, the asymptotic clearance rates for all three cases are much closer. Figure 6a–c also reveals that transients persist longer when *Pe* is smaller; for $Pe=10$, the transient is longest for oscillatory flow and shortest for steady flow, whereas for $Pe=0.1$ the opposite trend is true.

Figure 6d–f show the asymptotic profiles of the radially-averaged concentration $C_m$ for the three different flows at $Pe=\{10, 1, 0.1\}$. In all cases, the profile for oscillatory flow is linear throughout the domain. However, for steady and pulsatile flows, the profiles only approach a linear profile in the case of $Pe=0.1$; for $Pe=\{10,1\}$, the concentration saturates near $C_m=1$ for much of the domain with a rapidly decreasing radially-averaged concentration near the outlet ($z=0.25$ mm). For a fixed *Pe*, steady flows exhibit a larger fraction of the domain with $C_m=1$ saturation than pulsatile flows, and the saturated domain fraction for steady and pulsatile flows increases with *Pe*. Supplementary Videos 4, 5 and 6 provide animations of the solute concentration and radially-averaged concentration profiles $C_m$ for the three flows at $Pe=10$, $Pe=1$, and $Pe=0.1$, respectively.

## Discussion

We have presented the results of advection-diffusion simulations in cylindrical annuli with two configurations: a high-concentration bolus that spreads and solute transport from a source to a sink. The two configurations serve as idealized models of the transport of amyloid-$\beta$, other wastes, or CSF tracers such as dextran. The bolus simulations model a localized source, while the source-sink simulations approximate transport between high- and low-concentration regions. In both configurations, we simulated three different types of flow: steady, purely oscillatory, and pulsatile (a superposition of steady and oscillatory components). Our bolus-spreading simulations agree closely with prior analytic predictions. We found that the width of the bolus increases most rapidly, and the location of peak concentration travels most rapidly, in steady flow, followed by pulsatile flow, followed by oscillatory flow. We also found that the solute mass transported out of the PVS increases most rapidly in steady flow, followed by pulsatile flow, followed by oscillatory flow. Varying the Péclet number from 0.1 to 10, we found that higher values caused more rapid transport. In the source-sink configuration, in all cases, we found that solute is removed at a constant rate once an initial transient has passed. For all *Pe* tested, we found that steady flow caused the fastest solute removal, followed by pulsatile flow, followed by oscillatory flow, but for $Pe=0.1$ the asymptotic rates of clearance were much closer for all three flows than for $Pe=10$.

Our results use multiple measures to show that in both configurations a purely oscillatory flow of CSF in a PVS would remove wastes only slightly faster than diffusion alone. For a bolus of solute in purely oscillatory flow, there is no net axial transport in that the location of the peak concentration (or, equivalently, the solute center of mass) remains unchanged. Furthermore, a purely oscillatory flow enhances the effective diffusion little, whereas a flow with even a weak steady velocity component enhances the effective diffusion much more, rapidly spreading any local, high-concentration bolus. In fact, Fig. 5 demonstrates that a steady flow (black solid curve) with 1% the maximum velocity of an oscillatory flow (red dashed curve) will transport 50% of the solute 250 $\upmu$m downstream more rapidly. In physiological conditions, however, a localized bolus seems less likely than an influx of waste with concentration that is nearly uniform along the PVS. In such a case, the near-uniform concentration profile means enhanced diffusion will be an even less effective transport mechanism. Rather, clearance requires advective transport out the end of the PVS, which our source-sink simulations show to be achieved much more quickly by a steady flow component than by a purely oscillatory flow when $Pe\gtrsim 1$. In the low Péclet number case, we found that rates of solute transport for all three types of flow are similar, but steady flow still leads to the most rapid clearance.

Our findings for purely oscillatory flows can be compared to prior results from other studies focused on modeling penetrating PVSs. We find a maximum enhancement of the effective axial diffusion coefficient of only about 5%. In contrast, Asgari et al.^22^ report enhancement factors in the range of 27–110% (this was computed by evaluating Eq. (4) for $R_{\text{o}}$, assuming $R_{\text{s}}=0$, using values they reported in Table 2^22^). Although many of their parameters differ moderately from ours, the discrepancy is at least partially due to their much larger Péclet numbers, in the range $276 \le Pe \le 1380$ (computed using $D= 2$–10 $\upmu$m$^2$/s, $a=10\,\upmu$m, and $u_\text{max}=276\,\upmu$m/s). For our parameters, these Péclet numbers result in a very large enhancement factor ranging from 4000 to 99000%. However, such a large oscillatory $u_{\text{max}}$ (and therefore *Pe*) is unrealistic and would likely decrease substantially if realistic boundary conditions had been implemented^43^. Rey and Sarntinoranont^25^ developed an hydraulic network model of oscillatory CSF flow in a penetrating PVS and the neighboring parenchyma. For their model geometry and maximum reported PVS velocity, $u_{\text{max}}=30$ $\mu$m/s, our calculations suggest the enhancement factor would be only about 0.3% (using Pe = 4.4 based on $D=1.35\times 10^{-10}$ m$^2$/s). Sharp et al.^26^ perform a thorough analysis of dispersion due to oscillatory flow using a two-dimensional Darcy–Brinkman porous media model. For parameters similar to ours and an estimated PVS permeability of 1.8$\times 10^{-14}$ m$^2$, they find an enhancement factor of only 0.00012% for $Pe=3.14$. Jin et al.^44^ simulated solute transport in the brain parenchyma (not in PVSs) and found that including an oscillatory flow component had little effect, but including a steady component significantly accelerated transport.

In contrast to a couple of these prior studies of metabolite transport in PVSs^22,26^, we considered the fluid domain within a PVS to be an open space, rather than a porous medium. It is now known that the PVSs around surface (pial) arteries in the mouse brain are indeed open, not porous^36^, but the nature of PVSs around penetrating vessels is still undetermined. However, allowing for the different velocity profiles that would occur in Darcy-Brinkman flow in a porous annular channel, one would still expect to find differences in transport among steady, oscillatory, and pulsatile flows quite similar to those we find here for an open channel. Darcy flows have uniform effective velocity profiles in which no Taylor dispersion would occur; however, if the small-scale flow through pores was modeled, the effective diffusivity would be enhanced slightly due to Taylor dispersion and the disordered geometry. Numerical studies modeling dispersion in the spinal canal have provided evidence that trabeculae play a crucial role in dispersion enhancement^45–47^, suggesting that porous-medium continuum models may not always provide an accurate framework for predicting enhanced transport due to dispersion.

We did not simulate motions of the PVS walls, nor any other mechanism which would drive steady, oscillatory, or pulsatile flow. Instead, we prescribed known velocity fields for laminar flows in a channel at rest, driven by steady and oscillatory pressure gradients. We chose the domain geometry based on an in vivo image of a penetrating arteriole PVS^39^, which has a PVS cross-sectional area that is likely much larger than that of a venule^48^. While not representative of the precise fluid motions expected in penetrating PVSs, these flows do portray the general transport behavior one would expect. We implemented our simulations in a two-dimensional axisymmetric domain. Real PVSs are not perfect, concentric cylinders; rather, they are often elongated and/or eccentric^37^. Our simulations neglected azimuthal flow, although it has been shown to occur when an artery pulses in a non-axisymmetric PVS^28,49^. However, the azimuthal component is far weaker than the axial component, which was the focus of our simulations. Thus we expect that including three-dimensional boundaries and flow would cause only small adjustments to the effective diffusivity and mass efflux rates, and would not affect our central findings.

Our study was motivated by prior observations showing that flows oscillate in synchrony with the cardiac cycle in PVSs at the brain surface^9,10^. It has been suggested that there may be oscillations at the respiration rate, with or without oscillations at the heart rate^50^. In that case, our central conclusion would remain unchanged, since the respiration frequency is lower than the cardiac frequency and would introduce no new nonlinear effects: steady flows, even if slow, would transport solutes faster than oscillatory or pulsatile flows. In the PVS surrounding the spinal cord, however, nonlinear effects play an appreciable role and the situation is more complex^51,52^.

We did not simulate motions of the PVS walls, nor any other mechanism which would drive steady, oscillatory, or pulsatile flow. Instead, we prescribed known velocity fields for laminar flows in a channel at rest, driven by steady and oscillatory pressure gradients. We chose the domain geometry based on an in vivo image of a penetrating arteriole PVS^39^, which has a PVS cross-sectional area that is likely much larger than that of a venule^48^. While not representative of the precise fluid motions expected in penetrating PVSs, these flows do portray the general transport behavior one would expect. We implemented our simulations in a two-dimensional axisymmetric domain. Real PVSs are not perfect, concentric cylinders; rather, their cross-sections are often flattened and/or eccentric^37^. Our simulations neglected the radial and azimuthal motions shown to occur when an artery pulses in a non-axisymmetric PVS^28,49^. However, these radial and azimuthal velocity components are much weaker than the axial component, and diffusion dominates solute transport in the radial and azimuthal directions in all our simulations (because of the narrowness of the PVS), so if these radial and azimuthal motions were included, the solute concentration would still be essentially uniform over any cross-section, and the dispersion in the axial direction would be essentially unchanged. Also, secondary flows due to curvature of the PVS axis, which are an inertial effect, will be essentially absent at the low Reynolds numbers of PVS flows. Thus, we fully expect that including more realistic three-dimensional PVS configurations would cause only small adjustments to the effective diffusivity and mass efflux rates and would not affect our central findings.

## Supplementary information

Supplementary Information.

Supplementary Video 1.

Supplementary Video 2.

Supplementary Video 3.

Supplementary Video 4.

Supplementary Video 5.

Supplementary Video 6.
