# Author Correction: Dispersion as a waste-clearance mechanism in flow through penetrating perivascular spaces in the brain

**DOI:** 10.1038/s41598-021-99517-0

**Published:** 2021-10-01

**Authors:** Daniel E. Troyetsky, Jeffrey Tithof, John H. Thomas, Douglas H. Kelley

**Affiliations:** 1 Department of Mechanical Engineering, University of Rochester, Rochester, 14627 NY USA; 2 Department of Mechanical Engineering, University of Minnesota, Minneapolis, 55455 MN USA

Correction to: *Scientific Reports* https://doi.org/10.1038/s41598-021-83951-1, published online 25 February 2021

The original version of this Article contained a typographical error in Equation 10, where the last term in the numerator was incorrect.

10 $$\begin{array}{cc}{\kappa }_{13}& =\frac{1-3{\sigma }^{2}-2{\sigma }^{4}{\Sigma-1}}{8(1-{\sigma }^{2})},\end{array}$$

with $$\sigma ={r}_{1}/{r}_{2},\Sigma =(1-{\sigma }^{2})/\mathrm{ln}{\sigma }^{2},$$ and $$S=1+{\sigma }^{2}+2{\Sigma}^{2}$$.

now reads:

10 $$\begin{array}{cc}{\kappa }_{13}& =\frac{1-3{\sigma }^{2}-2{\sigma }^{4}{\Sigma }^{-1}}{8(1-{\sigma }^{2})},\end{array}$$

with $$\sigma ={r}_{1}/{r}_{2},\Sigma =(1-{\sigma }^{2})/\mathrm{ln}{\sigma }^{2},$$ and $$S=1+{\sigma }^{2}+2\Sigma$$.

The original Article has been corrected.

